# Retracted: Construction and Immunogenicity of DNA Vaccines Encoding Fusion Protein of Porcine IFN-*λ*1 and GP5 Gene of Porcine Reproductive and Respiratory Syndrome Virus

**DOI:** 10.1155/2022/9802362

**Published:** 2022-03-26

**Authors:** BioMed Research International

*BioMed Research International* has retracted the article titled “Construction and Immunogenicity of DNA Vaccines Encoding Fusion Protein of Porcine IFN-*λ* 1 and GP5 Gene of Porcine Reproductive and Respiratory Syndrome Virus” [1]. As raised on PubPeer [2], the article was found to contain discrepancies in Figures 2(a) and 2(b). Specifically, panels (a) and (b) in Figure 2 appear to be very similar, except for the presence/absence of the band in lane 1 at 2692 bp. There are also inconsistencies with the expected orientation of the bands.

The authors responded to explain that different shapes in the same lane may be due to the machine and electrophoresis, however, it was determined that this was not a satisfactory response, and the article is therefore being retracted with the agreement of the Editorial Board.

The authors agree to the retraction and the notice.
